# Distal Tibia Epiphysiodesis After Saphenous Vein Catheterization During Treatment for Prematurity

**DOI:** 10.7759/cureus.21596

**Published:** 2022-01-25

**Authors:** Panagiotis V Samelis, Panagiotis Kolovos, Christos Loukas, Eleni Sameli, Flourentzos Georgiou

**Affiliations:** 1 Orthopaedics, Children’s General Hospital Panagiotis & Aglaia Kyriakou, Athens, GRC; 2 Orthopaedics and Trauma, Apostolos Pavlos Trauma Hospital, Athens, GRC; 3 Operation Center, National Public Health Organization, Athens, GRC

**Keywords:** growth arrest, tibial osteotomy, picc line complication, growth plate injury, neonatal intensive care unit (nicu), extravasation injury, deformity, epiphysiodesis

## Abstract

Extravasation injuries are frequent complications, especially in extremely preterm neonates treated in neonate intensive care units (NICU). Depending on the type of the extravasated substance, the duration, and the amount of the leak, extravasation may result in necrosis of the soft tissues adjacent to the leak, compartment syndrome, and limb amputation. However, in some cases, the results of extravasation may be evident years after NICU treatment. In this case report, we describe a rare case of physeal arrest of the distal tibia in a preterm and discuss the possible causes.

## Introduction

Physeal arrest in children is usually the result of sepsis or trauma [1].

Sepsis is a major cause of morbidity in preterms (gestational age less than 37 weeks, 11% of all births) [2]. Immune dysfunction and absence of transplacentally acquired maternal IgG antibodies predispose to bacteremia and sepsis of the preterm [3]. Furthermore, preterms are usually treated in neonate intensive care units (NICU), where they undergo multiple invasive procedures, such as intubation, surgery, puncture, or catheterization of vessels or other organs [3]. These interventions breach the normal barrier of the skin and the mucous membranes against microbes and increase the risk of healthcare-associated infections (HAI) of the neonates [3,4].

Bloodstream infection, pneumonia, and meningitis are the most frequent HAI’s in NICUs, accounting for 73%, 12%, and 10% of cases respectively [4]. In another study, the most frequent HAI in NICU were pneumonia (64.9%), followed by urinary tract infection (20.3%), sepsis (9.5%), and omphalitis (5.4%) [5], but not osteomyelitis or septic arthritis. No HAI-associated osteomyelitis or septic arthritis was observed in a study of 4,615 neonates during NICU treatment in Brazil [6]. Extreme prematurity (gestational age ≤26 weeks), very low birth weight (birth weight ≤ 1500 g), and interventional procedures on the neonate increase the risk of HAI [4]. Roversi et al. stress that the incidence of neonatal osteomyelitis is far lower than the incidence in the overall pediatric population, which is 13 cases per 100,000 in developed countries [7]. In a retrospective series of 22 cases, they found that in 50% of neonate osteomyelitis an invasive procedure such as central vein or umbilical vein catheterization is recorded [7]. The most frequently affected sites for neonate osteomyelitis are the femur (40.9%) and humerus (36.3%), while the tibia (at the knee or ankle) is the least frequent location (18.2%) [7]. These locations are considered the result of hematogenous spread and not of direct inoculation of microbes from the invasive procedure per se [7]. Zhan et al mention that, due to non-typical presentation at early stages, if not suspected by the clinician, diagnosis and treatment of neonatal osteomyelitis may be delayed [8]. Once again, the authors state that, neonate osteomyelitis is not very frequent. Its incidence is estimated at only 1-3 cases per 1000 hospital admissions per year [8],

On the other hand, the frequency of extravasation injuries is considerably higher among preterms [9]. Extravasation injury is deemed any tissue injury caused after leakage of medication or fluids out of a peripheral vein catheter to the surrounding tissues [9]. While in adults extravasation has been reported in 5% of cases after administration of cytotoxic drugs [10], in neonates extravasation may be observed in 18%-46% of cases. In almost all cases (98%), extravasation injuries complicate peripherally inserted vein catheters [9]. The small size and the extremely fragile wall of the peripheral veins of the neonate, the prolonged intravenous therapy, the difficulty to stabilize catheters on the neonate, and the inability of the neonate to communicate pain are possible explanations [9]. Lower gestational age and low birth weight are particularly important risk factors for extravasation. Up to 70% of extravasation injuries are observed in extremely preterm infants [9].

Extravasation injuries usually affect soft tissues adjacent to the leak. In extreme cases, tissue injury may be irreversible and lead to compartment syndrome, tissue death, and amputation of the limb [9,10]. Mechanisms of tissue injury secondary to extravasation include direct cytotoxic effect of the leaking substance, osmotic pressure gradient across the cell membrane and cell death, ischaemic necrosis from vasopressors and cationic solutions, mechanical outer compression of arterioles and subsequent ischaemic necrosis, and finally, bacterial inoculation and local superinfection. These mechanisms may act solely or in concert [9].

Consequently, in neonates, physeal arrest may be part of the spectrum of extravasation injury [11-13].

We describe a rare case of varus deformity of the right tibia due to partial arrest of the distal tibial physis in a preterm girl. Concomitant monoplegia of the lower limb masked the underlying distal tibia physeal arrest, which, however, came to the surface with ongoing growth. The possible causes of this deformity are discussed.

## Case presentation

**Figure 1:**
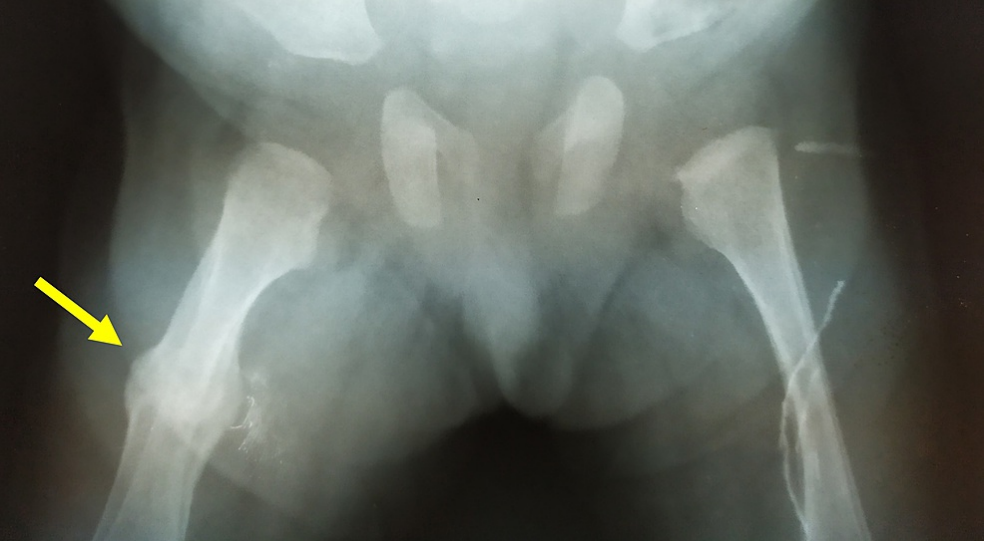
Birth-associated fracture of the right femur; sufficient callus formation at the age of two weeks. Arrow indicates the healed fracture of the right femur.

A 7-year-old girl presented with a varus deformity of her right distal tibia. She had a history of premature birth (third born, cesarean section, gestational age 26 weeks, birth weight 730 g) due to maternal pre-eclampsia. She was admitted to the NICU because of respiratory insufficiency and suspected necrotic enterocolitis. She received broad-spectrum antibiotics. She had a birth-associated femoral shaft fracture (Figure 1). After one month in the NICU, she developed bacteremia from Serratia, secondary to an inflammation of the left humerus (positive cultures in blood and pus), which was treated according to the antibiogram. Brain ultrasound showed an increased size of the left ventricle and grade 1 hemorrhage. While in the NICU, her parents report right saphenous vein catheterization, to facilitate systemic intravenous antibiotic administration. The patient was discharged from the NICU after three months.

**Figure 2:**
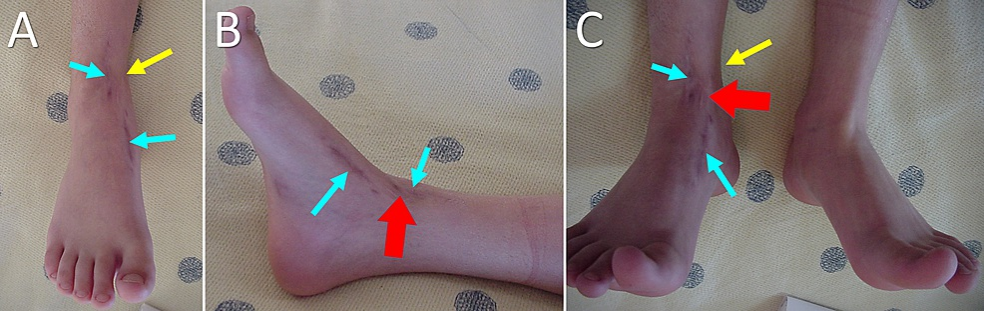
Varus deformity of the right distal tibia at the age of seven years. A. Front view of the right ankle, B. Medial view of the right ankle. C. Ankle dorsiflexion of the affected right and the healthy left lower limb. Yellow arrows indicate the level of the varus deformity of the right tibia. Blue arrows indicate the surgical scars of the soft-tissue procedure. Red arrows indicate irregular scar formation over the medial malleolus, compatible with antecedent skin necrosis.

**Figure 3:**
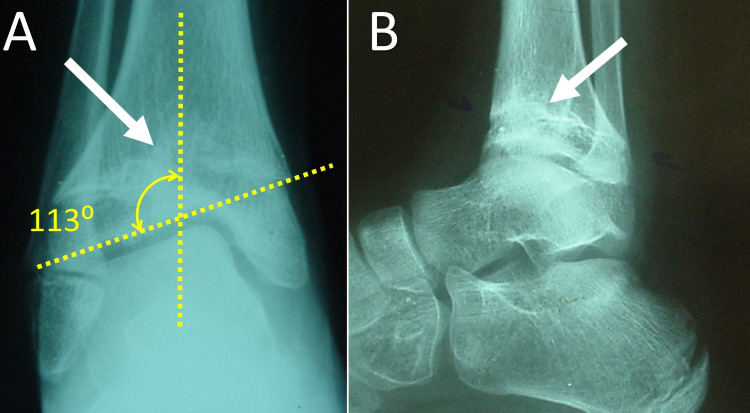
Anteroposterior (A) and lateral (B) plain X-ray of the right ankle joint of the patient at the age of seven years. A wide partial physeal bar (white arrow), causing central growth arrest of the distal tibial physis with subsequent angular deformity of the ankle joint (dotted lines) is evident

According to the patient's history, which was given by her parents, at about the age of five years, the patient presented an inwardly pointing right forefoot. No traumatic or septic history about the ankle joint could be recalled during the previous years. At the age of six years, the patient underwent a soft tissue procedure at the right ankle joint, due to concomitant monoplegia of the right lower limb. However, the deformity relapsed during the following months (Figure 2). Radiologic examination showed a partial epiphysiodesis of the distal tibia (Figure 3).

**Figure 4:**
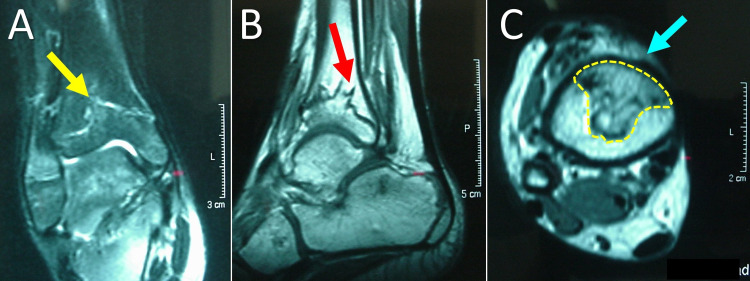
MRI at the level of the distal physis of the right tibia. A. Coronal view of the distal physis of the tibia: yellow arrow indicates the proximal extension of the physeal bar. B. Sagittal view: red arrow indicates the dorsal extension of the physeal bar. C. Transverse view: yellow interrupted line circumscribes the physeal bar. The physeal bar comprises more than 50% of the total surface of the growth plate. Note the anteromedial location of the base of the physeal bar, towards the medial malleolus (blue arrow).

Further assessment with right ankle MRI showed the presence of a physeal bar, which occupied more than 50% of the growth plate of the distal tibia (Figure 4). Physeal bar excision was contraindicated. Furthermore, the angular deformity of the tibial plafond was more than 20 degrees in varus, indicating the need for a correctional osteotomy, irrelevant of the extension of the bony bar [1].

**Figure 5:**
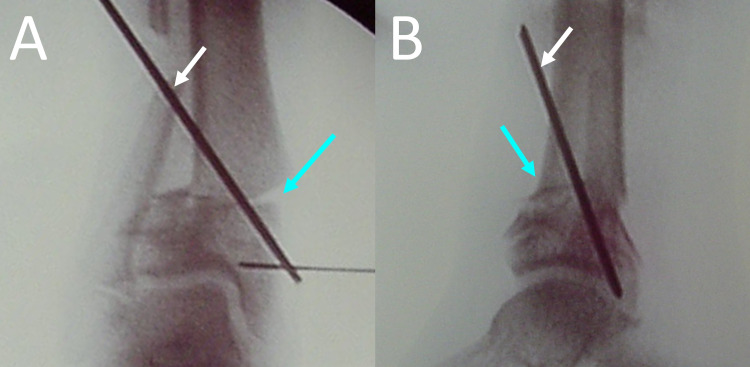
Anterolateral (A) and lateral (B) view of the supramalleolar right tibia osteotomy. Blue arrows indicate the supramalleolar osteotomy. White arrows indicate the percutaneous pin, which is used to stabilize the osteotomy.

A supramalleolar corrective osteotomy of the tibia with concomitant resection of 1 cm of the distal third of the fibula was decided. The removed segment of the fibula was used to fill the gap of the tibial osteotomy, after correction of the deformity. The tibial osteotomy was stabilized using a percutaneous pin (Figure 5). The parents were informed of future limb shortening and the need for additional surgery. 

**Figure 6:**
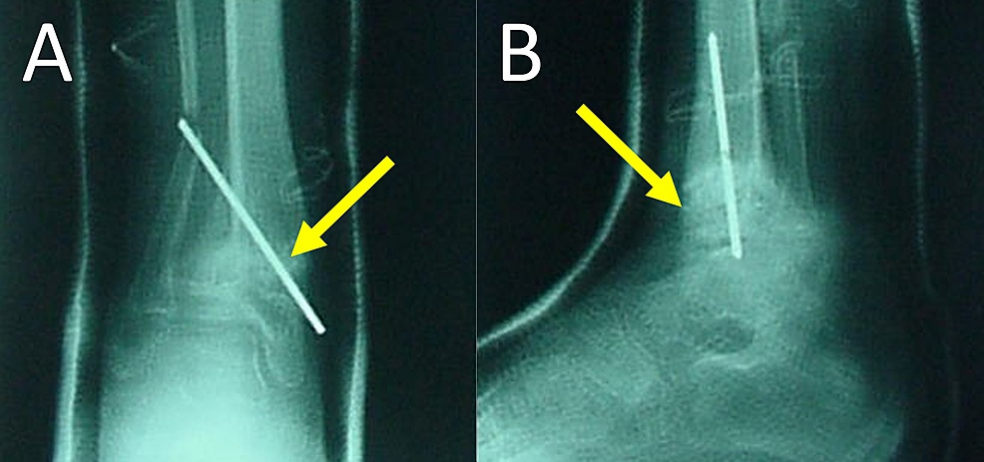
Anteroposterior (A) and lateral (B) radiography of the right ankle joint at two months postoperatively. Arrows indicate the healed supramalleolar osteotomy

After two months, the osteotomy had healed and the pin was removed (Figure 6).

At the age of 15 years, the patient underwent distraction osteogenesis of the right tibia to correct limb length discrepancy of 5 cm due to a shorter right tibia. A unilateral frame was used and a proximal tibial osteotomy was selected. The process was completed after two years and the frame was removed. 

**Figure 7:**
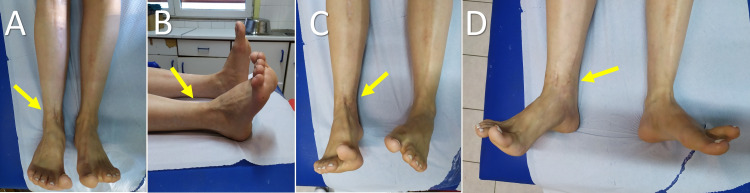
Clinical presentation of the lower limbs at the age of 27 years. Arrows indicate the affected limb. A. Front view, B. Lateral view. C. Inversion, D. Eversion

**Figure 8:**
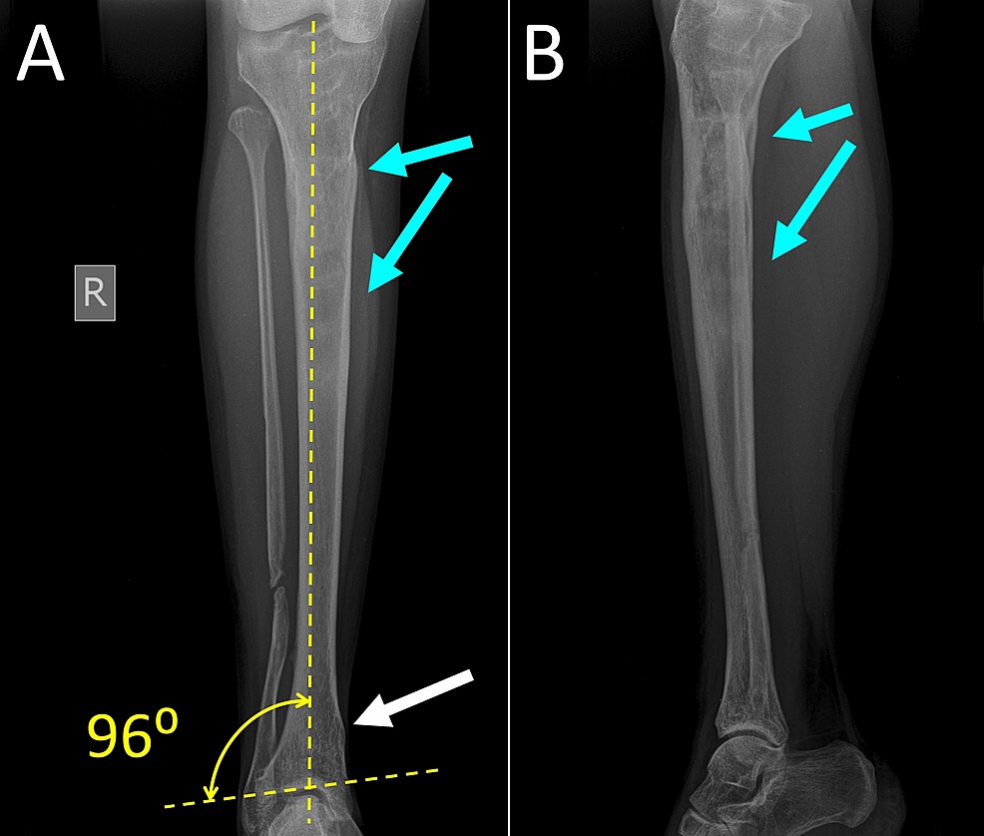
Anteroposterior (A) and lateral (B) radiography of the right tibia at the age of 27 years. White arrow indicates the location of the supramalleolar osteotomy. Dotted lines depict the final correction of the deformity of the right ankle. Blue arrows indicate the location of the distraction osteogenesis of the right tibia, which was performed at the age of 15 years.

The patient was re-examined after 20 years. Her foot and ankle are pain-free. She has a normal bone mass index (19.6 kg/m2) and a normal gait pattern. She does not complain of any restrictions during daily living or working, due to her right tibia medical history. On clinical examination, mild restriction of right ankle extension and flexion and mild restriction of right foot inversion, compared to the contralateral side, were manifested (Figure 7). On radiology, the right tibial plafond was in slight varus, however, no signs of arthrosis or instability were evident (Figure 8).

## Discussion

Very few cases of physeal arrest of the distal tibia secondary to extravasation from peripheral vein catheterization in neonates have been described in the literature.

Sanpera et al. presented two neonates, which had a history of extravasation after saphenous vein catheterization. Both children were referred to the orthopedic department at the age of four, because of a deformed (varus or equinovarus respectively) and shortened tibia (minus 2 cm compared to the contralateral limb). The authors suggest three mechanisms of physeal arrest after extravasation injury [11]. First, soft tissue necrosis leads to local scar formation, which tethers the adjacent part of the growth plate [11]. Second, local sepsis after tissue necrosis may destroy the proliferating layer of the growth plate. Third, necrosis of local perichondrium may lead to bony bar formation and local growth arrest [11]. They explained, why the deformity was evident four years after the extravasation and not earlier. They suggested, that the secondary ossification center of the distal tibial epiphysis is very small or absent in the neonate, and compensates to some point for the partially damaged physis. However, when the ossification center grows until it touches the physis, no further growth is possible and the bony bar of the physis becomes evident [11].

Fullilove et al. presented three preterms with deformity and shortening of the distal tibia secondary to extravasation injury after saphenous vein catheterization. Osseous interventions (osteotomy, lengthening) were performed after the age of six years [12].

Wada et al. published one case of physeal arrest of the lateral malleolus and the lateral part of the distal tibial physis in a term-born male neonate, secondary to extravasation necrosis from vein catheterization on the dorsum of the foot [13]. At the age of six, the boy had a valgus deformity of the ankle and a shorter tibia. At this age, he underwent a reconstruction osteotomy of the ankle to address axial deformity. At the age of eight, the shorter tibia was lengthened by 6 cm. At the age of 12 years, an additional osteotomy was necessary to correct the recurrence of ankle valgus [13].

We present a preterm female neonate with partial distal tibial physis arrest and concomitant monoplegia of the right lower limb. The patient was a high-risk candidate for all complications associated with prematurity, such as primary bloodstream infection, pneumonia, meningitis, neurologic disease, etc [3,6]. On the other hand, according to published data, the risk for neonatal osteomyelitis (acute or HAI) is very low [6-8]. Furthermore, in neonates, osteomyelitis of the metaphysis quite frequently (up to 76% of cases) coexists with epiphysitis or septic arthritis, due to vascular communication of the metaphysis and the epiphysis in neonates [14,15]. In the presented case, the articular cartilage of the ankle joint had no radiological signs of infection, which supports the non-septic mechanism of physeal arrest. Thus, the history of saphenous vein catheterization and the high incidence of extravasation injury in preterms [9] render extravasation injury the prevailing mechanism of physeal arrest in this particular case.

In the presented case, the deformity became evident after the age of five years. This agrees with the published case reports of ankle epiphysiodesis secondary to extravasation in neonates [11-13].

Limb deformity and shortening is a well-recognized complication of femoral, brachial, or radial artery catheterization [16,17]. Growth arrest in these cases is attributed to ischaemic embolization of the growth plates of the limb distal to the site of catheterization [16,17]. On the contrary, limb shortening after peripheral venous catheterization seems to be extremely rare. The published literature is confined to the papers mentioned above [11-13]. Perichondral and growth plate injury secondary to extravasation from peripheral vein catheters seem to be the most possible mechanism for the osseous deformity of the presented case [9].

## Conclusions

Physeal arrest is a rare but existing sequel of extravasation injury after peripheral vein catheterization in neonates. Deformity and limb shortening may be evident several years after the initial injury. In the early stages of the physeal arrest, the immature secondary ossification center of the distal tibial epiphysis and comorbidities, such as monoplegia, may mask the underlying pathologic growth until early childhood.
